# Image-based *in vivo* assessment of targeting accuracy of stereotactic brain surgery in experimental rodent models

**DOI:** 10.1038/srep38058

**Published:** 2016-11-30

**Authors:** Janaki Raman Rangarajan, Greetje Vande Velde, Friso van Gent, Philippe De Vloo, Tom Dresselaers, Maarten Depypere, Kris van Kuyck, Bart Nuttin, Uwe Himmelreich, Frederik Maes

**Affiliations:** 1 Department of Electrical Engineering (ESAT/PSI), KU Leuven & Medical Imaging Research Center, University Hospital Leuven, Leuven, Flanders, Belgium; 2 Molecular Small Animal Imaging Center (MoSAIC), Faculty of Medicine, KU Leuven, Leuven, Flanders, Belgium; 3 Biomedical MRI unit, Department of Imaging and Pathology, Faculty of Medicine, KU Leuven, Leuven, Flanders, Belgium; 4 Laboratory for Experimental Functional Neurosurgery, Department of Neurosciences, Faculty of Medicine, KU Leuven, Leuven, Flanders, Belgium

## Abstract

Stereotactic neurosurgery is used in pre-clinical research of neurological and psychiatric disorders in experimental rat and mouse models to engraft a needle or electrode at a pre-defined location in the brain. However, inaccurate targeting may confound the results of such experiments. In contrast to the clinical practice, inaccurate targeting in rodents remains usually unnoticed until assessed by *ex vivo* end-point histology. We here propose a workflow for *in vivo* assessment of stereotactic targeting accuracy in small animal studies based on multi-modal post-operative imaging. The surgical trajectory in each individual animal is reconstructed in 3D from the physical implant imaged in post-operative CT and/or its trace as visible in post-operative MRI. By co-registering post-operative images of individual animals to a common stereotaxic template, targeting accuracy is quantified. Two commonly used neuromodulation regions were used as targets. Target localization errors showed not only variability, but also inaccuracy in targeting. Only about 30% of electrodes were within the subnucleus structure that was targeted and a-specific adverse effects were also noted. Shifting from invasive/subjective 2D histology towards objective *in vivo* 3D imaging-based assessment of targeting accuracy may benefit a more effective use of the experimental data by excluding off-target cases early in the study.

Stereotactic neurosurgery is used to introduce a needle or an electrode at a precise location in the brain, for instance to perform deep brain stimulation (DBS), lesioning (e.g. ablation) or cell-based therapy in patients to ameliorate symptoms of neurological disease. Clinical use of stereotactic neurosurgery benefits from experimental research on rodent models, for instance to identify potential target regions. Likewise, small animal investigations like *DBS* for neuromodulation^1^,^2^, *electrochemical/ electrolytic lesioning* for creating animal models or ameliorating symptoms^3^,^4^, as well as *gene therapy* investigations involving injection of stem cells/therapeutic agents/tracers^5^,^6^,^7^ requires stereotactic brain surgery. They rely on standard stereotaxic brain atlases (e.g. The rat brain in stereotaxic coordinates (George Paxinos and Charles Watson, Academic Press, 2006^8^) and skull landmarks (e.g. bregma (B), lambda (L)) for localizing the target in the rodent brain and for surgery planning^9^. Stereotaxic frames used in small animal brain surgery have species-specific head mounting platforms, with a manipulator that can move in three dimensions along the axes with respect to the head holder. The targeting accuracy of such devices is dependent on numerous factors, such as inter-animal anatomical variability, positioning errors (e.g. skull flat position), scaling errors (e.g. when using the same atlas with animals of a different size or strain), errors in intra-operative localization of bregma, operator-specific deviations etc. Some of these sources of inaccuracy in small animal stereotactic surgery have been characterized in previous reports^10^,^11^,^12^,^13^.

Assessment of targeting accuracy in rodent brain is predominantly done by *ex vivo* histology. By identifying the location of the electrode trace on a two-dimensional (2D) histological section and mapping this section onto the corresponding slice of a representative stereotaxic brain atlas, a schematic representation of the electrode tip locations in different animals can be reconstructed as illustrated in Fig. 1. Various studies involving different targets (e.g. subthalamic nucleus, substantia nigra, hypothalamus…) have reported that the actual electrode tip location in different animals was dispersed across different sub-sections of the target region or even outside this region despite having identical entry and target coordinates defined for all animals^1^,^14^,^15^,^16^,^17^,^18^. For instance, while targeting the parafascicular nucleus (PF) of the thalamus, Vale-Martínez, *et al*.^14^ report about 50% (22 out of 42) targeting accuracy, i.e. electrode tip anywhere within PF, but the electrode tip locations are dispersed within the target, in spite of identical entry coordinates and angle of entry for all trajectories. Such targeting variability is typically accounted for by including a large number of animals. In case the electrode tip is found to be outside the target region, the results from those animals are often discarded^19^.

The current practice of verifying targeting accuracy from 2D histological sections has several limitations. First of all, it relies on manual alignment of the histological sections with the stereotactic atlas, as illustrated in Fig. 1(b), which is necessarily only approximate and whereby any possible mismatch (due to e.g. slice plane inclination, shrinkage, distortion) is often ignored or underestimated. Furthermore, it is typically confined to a visual assessment of the location of the electrode tip with respect to the target region based on few 2D cross-sections only, without objective quantification of 3D targeting accuracy. Moreover, the 3D electrode trajectories and possible deviations from the planned trajectory (e.g. inclination) are difficult to assess from 2D histology and are therefore usually not evaluated. Also, whether the trajectory has damaged any critical structures (e.g. vasculature) along its path is not always reported. Finally, by relying on end-point histology to identify off-target subjects or those with possible deleterious effects from the surgery, valuable time and resources may have been lost, for instance in case of longitudinal studies including behavioral tests. If such studies are conducted over weeks to months, histological verification of whether the specific site of interest was successfully targeted at the time of surgery may become problematic. Thus, the conventional histological assessment of targeting accuracy is essentially a manual procedure that is tedious, error-prone and often also inconclusive and inadequate.

In a clinical setting, neuroimaging plays a critical role in the planning of stereotactic neurosurgery in humans. The availability of patient-specific pre-operative images facilitates accurate targeting and assists in minimizing deleterious injury of critical structures during surgery^20^,^21^,^22^. It is also routine clinical practice to acquire post-operative images in order to assess the presence of surgery induced intracerebral hemorrhage (ICH) and to verify that the actual trajectory did not deviate from the one as planned. For DBS, the electrode tip locations are also quantitatively assessed for possible offsets from the targeted neuromodulation site^23^,^24^.

With the advent of dedicated small animal imaging instrumentation, resolving the challenges in assessing targeting accuracy of stereotactic neurosurgery in preclinical research is gaining attention. The need and benefits of image-based pre-operative surgery planning in small animals has been previously reported in several studies^25^,^26^,^27^,^28^,^29^. However, several challenges still remain with respect to using imaging for prospective planning and/or retrospective assessment of stereotactic neurosurgery in animal models. For instance, access to methods and instrumentation (e.g. robotic guidance described in refs 28,29) for transferring the image-based planning to the animal is limited. Non-invasive visualization of the electrode using post-operative imaging has been previously reported (e.g.^30^). However, reconstruction of the electrode trajectory from *in vivo* images and quantification of targeting accuracy have not been widely investigated. This typically requires (a) acquisition of multi-modal (e.g. MRI, CT) *in vivo* rodent brain images, (b) fusion of intra-animal 3D images and their spatial normalization to a stereotaxic atlas, (c) 3D reconstruction of the electrode trajectory from the images, (d) localization of the electrode tip with respect to anatomical targets, (e) quantification of targeting accuracy, and (f) reporting image-based evidence of possible deleterious effects of the surgery, e.g. vascular damage.

In this paper, we present a multi-modal imaging approach (using MRI and CT) for retrospective assessment of stereotactic interventions in small animal models. We report on the workflow and quantification methods and provide proof of concept by applying our method to assess stereotactic electrode insertion for two different neuromodulation targets in rats. We demonstrate the feasibility and discuss the potential benefits of *in vivo* assessment of stereotactic neurosurgery in rodent brain.

## Materials and Methods

### Experimental setup

Two experiments (Exp.1 and Exp.2) with total fifteen (in Exp.1) and five (in Exp.2) adult male Wistar rats with a body weight (BW) of 291.6 ± 27.0 g (mean ± standard deviation (s.d.)) and 287.8 ± 8.0 g, respectively were conducted in this study to assess targeting accuracy.

For Exp.1, we mimicked the typical lesioning or injection procedures^1^,^4^,^5^,^6^,^7^ that involve stereotactic insertion/retraction of an electrode or needle at a predefined location in the brain. The resulting trace of the electrode/needle was used to assess the surgical accuracy as well as any deleterious injury in the brain. This is similar to the conventional histological procedure, albeit a post-operative *in vivo* imaging-based non-destructive 3D measurement. To this end, all animals were first subjected to *in vivo* imaging with CT and MRI on consecutive days. About one week later, the animals were stereotactically operated as described further. The electrodes were retracted immediately after insertion. In order to reconstruct the corresponding trace of the insertion, post-operative *in vivo* MR imaging was performed at the same day of surgery. The animals were subsequently sacrificed and prepared for histological assessment of the trace.

In Exp.2, we demonstrate the feasibility of *in vivo* imaging for both DBS (with electrode implanted in the brain) and lesioning/injection (electrode or needle inserted and retracted) experiments. Immediately after the surgery, a post-operative *in vivo* CT of the rodent brain with implanted electrode in place was acquired. The electrode was subsequently removed and a post-operative *in vivo* MR image was recorded to assess targeting accuracy based on the trace generated by the electrode. By co-registering the two post-operative *in vivo* images, i.e. CT and MRI, the agreement between the physical electrode in CT and its trajectory as reconstructed from its trace in MRI was verified. Moreover, adverse effects were also documented, solely based on the post-operative imaging data.

It is important to note that the pre-operative MRI/CT images from Exp.1 were not considered for prospective planning or intra-operative guidance in this study. The reasons being that *in* vivo rodent brain MRI provides insufficient image resolution and contrast to prospectively identify animal-specific subnucleus structures for targeting. Moreover, the reliability of intra-operatively localizing the same cranial landmarks that were identified in pre-operative CT requires prior investigation on their own. Hence, we confined the scope of the current study to retrospective assessment and thereby used the pre-operative images as mere baseline scans to resolve any likely ambiguities that may confound the assessment of post-operative images (e.g. electrode trace versus vessel or hemorrhage).

All the experimental methods, including *in vivo* imaging and stereotactic surgery were carried out in accordance with approved guidelines, in compliance with national and European regulations (European Directives 86/609/EEC, 1999/575/EC and 2010/63/EC), with approval by the KU Leuven Ethical Committee for Animal Experimentation (ECD, 123/2011).

### Pre-operative imaging

A pre-operative CT image (CTpre, only for Exp.1) of the head was acquired using an *in vivo* small animal micro-CT scanner (Skyscan 1076, Bruker microCT, Kontich, Belgium) at 35 μm voxel resolution (49 kV, 200 μA, 0.5 mm Al Filter, rotation step of 0.8°, 2 projections per rotation step, 180 ms exposure, 11 min total scanning time).

MR imaging was performed using a 9.4 Tesla small animal MR scanner (Biospec, Bruker Biospin, Ettlingen, Germany). A transversal T_2_^*^-weighted 3D FLASH anatomical scan of the brain was acquired pre-operatively (MRIpre, only for Exp.1) (FOV = 30 × 22 × 13 mm, resolution of 117 × 153 × 148 μm, TR/TE = 150/12 ms, flip angle = 30°, cross coil setup using a 7 cm linearly polarized resonator for transmission and an actively decoupled 3 cm surface coil for receiving, 12 minutes total scanning time).

Prior to MR and CT imaging sessions, animals were anesthetized in an induction chamber using a mixture of 3–4% isoflurane in 100% oxygen. During imaging, anesthesia was maintained with a mixture of 2% isoflurane in 100% oxygen. Respiration and body temperature were monitored throughout the acquisitions and maintained at 70 ± 15 breaths/min and 37 ± 2 °C, respectively.

### Surgery planning

In this study, two commonly used neuromodulation targets were considered for stereotactic intervention: (1) substantia nigra, compact part, dorsal tier (SNCD), a neuromodulation target in disease models of Parkinson’s Disease^31^ and (2) parafascicular nucleus (PF), a thalamic nucleus associated with psychiatric and neurological diseases^14^. Following conventional practice, the 3D stereotaxic coordinates of these targets were identified based on the rat brain atlas^8^ and defined as offsets relative to bregma along the medio-lateral (ML, left to right), anterior-posterior (AP, head to feet) and dorso-ventral (DV, depth from dorsal dural surface) axes. Planned target coordinates (T_plan_) were kept fixed for all animals, i.e. no animal-specific or image-based planning was considered. The planned trajectory of the electrode to the target was defined to be either straight vertically oriented (i.e. parallel to the DV axis (mid-sagittal plane), α_plan_ = 0°) or laterally inclined from the mid-sagittal plane (negative α) over an angle of about 20 degrees (i.e. α_plan_ ~ −20°). All trajectories were planned to be parallel to the mid-coronal plane (i.e. β_plan_ = 0°, no inclination in anterior-posterior (negative β) or posterior-anterior (positive β) direction). Given the target coordinates (T_plan_) and the orientation of the planned trajectory (α_plan_, β_plan_), the coordinates of the entry point (E_plan_) on the dural surface and its distance to the target (D_plan_) along the trajectory were derived.

In total, 23 electrode trajectories that targeted either SNCD or PF were planned in the 20 animals (17 unilaterally, 3 bilaterally) as summarized in Supplementary Table S1. Trajectories SNCD_1_, SNCD_2_ and SNCD_3_ targeted SNCD at different angles (α_plan_ = 0°, −18°, −21°), while trajectories PF_1_ to PF_4_ were all vertical (α_plan_ = 0°) and targeted anterior or posterior regions of PF nucleus.

### Stereotactic surgery

The stereotactic surgery procedure used to target SNCD and PF in the rodent brain was similar to the one previously described^1^. The anesthetized rats (intra-peritoneal injection of 22.5 mg/kg BW ketamine hydrochloride (Eurovet, Belgium) and 0.15 mg/kg BW medetomidine (Kela, Belgium)) were placed in a stereotactic head frame (0.1 μm resolution, Stoelting/David Kopf, Germany). The skull was exposed via a midline incision. The skull flat position was achieved by placing bregma (B_surgery_) and lambda (L_surgery_) in the same horizontal plane by adjusting the vertical tooth bar. A single burr hole was made at the planned entry location. Next, a single wired, insulated stainless steel electrode (Plastics One, Roanoke, VA, USA) with a 200 μm diameter, was mounted on the stereotactic arm, positioned at bregma and then translated antero-posteriorly and medio-laterally according to the planned trajectory (E_plan_, α_plan_, with bregma as origin). If the electrode appeared to be skewed after fixing it to the device, it was manually and visually (i.e. approximately) straightened first by the operator prior to insertion. Finally, after perforation of the dura mater with a 26 G needle, the electrode was lowered in a single motion according to the planned trajectory (D_plan_, with the dural surface as origin). For Exp.1, the electrode was left a few seconds in place before it was retracted and the scalp was subsequently sutured. The animals were subcutaneously administered with 1 mg/kg BW post-operative analgesic, meloxicam (Metacam, Boehringer Ingelheim, Belgium) and were allowed to recover prior to post-operative imaging. For Exp.2, the animals were operated, imaged, and euthanized under the same anesthesia session. The electrode was left in place within the brain and secured to the mechanically roughened skull with a minimal amount of UV-curing dental cement (Adhese Universal, Ivoclar Vivadent, The Netherlands). This minimal cement fixation allowed for easily removing the electrode after CT scanning with minimal stress/injury to the live animal and minimized CT/MR image distortion from dental cement and anchor screws.

### Post-operative imaging and histology

The animals were imaged with MRI few hours after the surgery (MRIpost) using the same 3D FLASH sequence with identical parameters as used for pre-operative MR imaging. For both experiments, the electrode was removed prior to post-operative MRI. In case of Exp.2, post-operative MRI was preceded by post-operative CT (CTpost) acquisition, with electrodes still inserted in the brain. Post-operative CT imaging parameters were adapted for imaging implanted metal electrodes: ~35 μm voxel resolution, 70 kV, 140 μA, 1 mm Al Filter, rotation step of 0.6°, 1 projections per rotation step, 200 ms exposure, 7 min total scanning time.

Histological assessment of targeting accuracy was performed for the animals of Exp.1. After post-operative imaging, the animals were euthanised by an overdose of Nembutal**®** (Sanofi, Brussels, Belgium) and fixated with 4% paraformaldehyde. The brain was then removed, postfixed for 24 hours in the same fixative and embedded in paraffin. Serial coronal sections of 75 micron thickness were produced using a vibratome (HM 650 V, MICROM International, Walldorf, Germany). Only sections within which the electrode trace could be observed were stained with either Nissl or hematoxylin and eosin (HE). Their AP distance from anterior commissure was noted to determine the site of implantation. The sections were examined using light microscopy (Leica DMLa, Leica CTRMIC, Germany) and digital images were captured. These histological images were visually matched to the corresponding coronal planes of the stereotaxic atlas^8^, based on anatomical landmarks. The approximate position of the electrode tip was visually localized in the histological images and its coordinates in stereotaxic space were reported as the targeted location as determined by histological assessment (T_hist_).

### Evidence of vascular damage

The post-operative MR images were visually inspected for evidence of intracerebral hemorrhage (ICH) as indication of vascular damage induced by the surgery. The presence of ICH was evaluated case by case based on a qualitative assessment of the apparent spread of the area of hypo-intense contrast around the visible trace of the electrode trajectory on the T_2_^*^-weighted MRIpost image. When available (Exp.1), the corresponding T_2_^*^-weighted MRIpre image was used as a baseline for comparison. Otherwise (Exp.2) the contralateral hemisphere without electrode/ insertion was used as reference.

### Spatial normalization of multi-modal images to stereotaxic atlas

Combining the anatomical information provided by the different imaging modalities pre- and post-operatively for the same animal (MRIpre, CTpre, MRIpost, CTpost), requires spatial alignment or fusion of all images. In addition, in order to compare electrode trajectories in different animals within the Paxinos coordinate space, the images of all animals have to be spatially normalized to this reference space. The T_2_-weighted average template at 25 μm isotropic resolution from the publicly available 3D MR histology (MRH) atlas of the Wistar rat brain that was developed by Johnson *et al*.^32^ was used for this purpose.

Multimodality image fusion and image-to-template spatial normalization were accomplished using an in-house developed, largely automated small animal image analysis pipeline^33^. The pipeline performs foreground extraction (i.e. a dilated brain mask with skull information), MRI inhomogeneity correction (by information minimization^34^) and image registration (by maximization of mutual information^35^). Identical parameter settings were used for all images for each processing step. In Exp.1, images of each animal were first aligned to the pre-operative MR image of the same animal (MRIpre) using a rigid (CTpre) or affine (MRIpost) transformation. In Exp.2, the post-operative MRI (MRIpost) was used as reference to which the corresponding CTpost was registered in a rigid manner. The MRIpre/ MRIpost images of different animals were subsequently spatially normalized to the MRI template of the atlas using a rigid transformation, conforming to the fact that no animal specific surgery planning (e.g. scaling) was performed. Both transformations were combined to transfer pre- and post-operative image coordinates of each individual animal into the common coordinate space of the MRH template, further denoted as MRIpax.

To determine the stereotaxic coordinates of the anatomical targets in the MRH Wistar rat template, the origin of the AP-ML-DV axes needs to be defined. The cranial landmarks of an original MRH image from Johnson *et al*.^32^ showing the skull and the cranial sutures, were used for this purpose. The bregma (B_mrh_) and lambda (B_mrh_) coordinates surveyed on this image were transformed to the MRH template by rigid registration.

In order to localize the electrode tip with respect to the anatomical target region of interest, the boundary of the target region needs to be identified. The contrast and spatial resolution of the *in vivo* MR images in our study proved to be insufficient to allow proper delineation of the small subnucleus targets (SNCD, PF). These structures are also not part of the MRI atlas of Johnson *et al*.^32^. Hence, we manually extracted their outlines from the coronal planes of the digital atlas of Paxinos and Watson^8^ and overlaid these on the images in the MRIpax space, such that the target region and the electrode trajectory could be jointly visualized on the post-operative MR images.

### Cranial landmarks

The skull landmarks bregma (B_ct_) and lambda (L_ct_) of the individual animals were manually indicated in the pre-operative CT images and their coordinates transferred into the common space of the atlas (MRIpax). Anatomical variability for our study population was assessed based on the bregma-lambda distance. The variability in the study population and/or possible bias in the spatial normalization of the images to the atlas was assessed by the offsets of the individual bregma and lambda points with respect to those of the MRH template (i.e., B_ct_ – B_mrh_; L_ct_ – L_mrh_). Assuming that the B-L plane of the MRH template is in skull flat position (defined by γ = 0°), the angular deviation of the corresponding B-L plane of the individual animals as measured in CT was quantified (i.e., γ_ct_). These offsets were tested for statistical significance by a one-sided paired t-test against zero mean (significance level = 0.05).

### 3D reconstruction of the electrode trajectory in MRI/ CT

**Figure 2:**
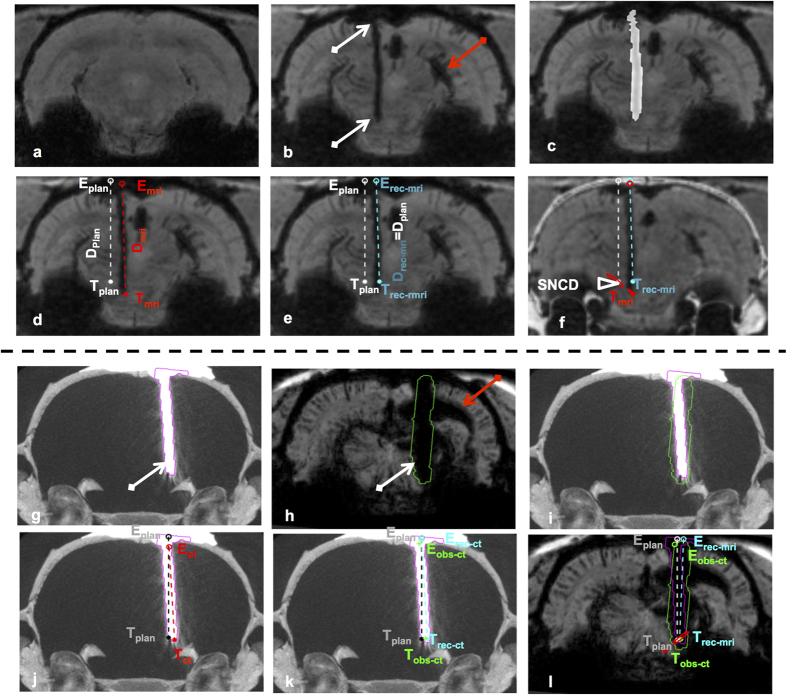
Reconstruction of the electrode trajectory from post-operative MRI and/or CT. (**a**) mIP of a coronal cross-section of a pre-operative T_2_^*^-weighted image; (**b**) mIP of the corresponding cross-section of the post-operative MR image of the same animal, showing the trace of the electrode in the left hemisphere as hypo-intense voxels (white arrows). The hypo-intense cluster in the right hemisphere (red arrow) appears to be an adverse effect caused by a second electrode (not seen in this cross-section); (**c**) Manual segmentation of the electrode trace (in white); (**d**) Planned trajectory (white line) with planned entry and target locations (E_plan_, T_plan_) as derived from the stereotaxic atlas, and actual trajectory (red line) with actual entry and target locations (E_mri_, T_mri_) as determined from post-operative MRI based on the segmented trace shown in (**c**). T_mri_ is at a depth D_mri_ from dura along the trajectory; (**e**) Reconstructed trajectory (cyan line) with the same orientation as in (**d**) but assuming that the entry point (E_rec-mri_) is at the level of dura as derived from the atlas and the target point (T_rec-mri_) is at the planned depth from dura along the trajectory (D_rec-mri_ = D_plan_); (**f**) Pre-operative CT with skull information overlaid on the post-operative MRI (a single slice instead of mIP as in **a**–**e**), with co-localization of the contour of the anatomical region being targeted (SNCD, in red); (**g**) MIP of a post-operative CT with implanted electrode. (**h**) mIP of corresponding MRIpost after electrode was removed. (**i**) MIP of CTpost with co-localization of the segmentation of the electrode from CTpost (pink contour) and its corresponding segmentation from the trace in MRIpost (green). Panels (**j**,**k**) show the trajectory with entry and tip positions from CTpost obtained from the electrode segmentation (**j**, in red, E_ct_, T_ct_), reconstructed based on D_rec-ct_ = D_plan_ (**k**, in cyan, E_rec-ct_, T_rec-ct_) and manually indicated by one observer (**k**, in green, E_obs-ct_, T_obs-ct_). The same trajectory as reconstructed from MRI (**l**, in cyan E_rec-mri_, T_rec-mri_) shows a good agreement at the tip location between CTpost, MRIpost and the observer.

For the visualization of the physical electrode as well as its trace, minimum intensity projection (mIP) of MRI and/or maximum intensity projection (MIP) of CT at the electrode location were used. To reconstruct the electrode trajectory from the post-operative MR images, its hypo-intense trace as visible in the MRIpost image was first manually delineated as illustrated in Fig. 2(b,c,h). When available, the corresponding pre-operative MR images in Fig. 2(a) were used as baseline reference to exclude possible confounds (e.g. blood vessels). For CTpost images, the hyper-intense contrast corresponding to the electrode was semi-automatically segmented by defining a cuboid region of interest around the electrode and applying an intensity threshold (I_max-ct_). The resulting segmentation is shown in Fig. 2(g). Additionally, to exclude any residual dental cement filling from biasing the determination of the electrode trajectory, the segmentation of the electrode in the CTpost image was eroded from the entry in the DV direction by few voxels (0.7 mm). The 3D orientation (α_plan_, β_plan_) of the trajectory was subsequently determined by computing the principal axis of the segmented electrode contrast/ trace using principal component analysis (PCA). The entry point (E_mri_, E_ct_) and the location of the electrode tip (T_mri_, T_ct_) along this trajectory were first determined from the extents of the segmented electrode trace as illustrated in Fig. 2(d,j). However, this was sometimes not reliable because of image ambiguity due to limited contrast and imaging artifacts, especially at the electrode tip or entry. To resolve this for both MRIpost and CTpost observations, we additionally determined a reconstructed entry point (E_rec-mri/rec-ct_, subscript indicates whether the reconstruction was from MRI (rec-mri) or CT (rec-ct)) by transforming the principal axis into the MRIpax space and finding its intersection with the dural surface of the Paxinos template (i.e. entry point at dura). The corresponding tip position T_rec-mri/rec-ct_ was located at a distance D_rec-mri/rec-ct_ = D_plan_ as defined by the planning along the reconstructed trajectory from E_rec-mri/rec-ct_, i.e. assuming no inaccuracy on the depth of the electrode once the orientation of its trajectory and its entry point were specified (see Fig. 2(e,k)). As a ground truth for CTpost observations, an observer manually picked the entry and tip positions for each trajectory in post-operative CT image (E_obs-ct_, T_obs-ct_ seen as green colored markers in Fig. 2(k,l)). These were then used to validate the semi-automatic PCA-based localizations in CTpost. Finally, overlaying the contours of SNCD or PF regions from Paxinos and Watson^8^ on the study images normalized to MRH template allows to determine whether the electrode tip is within or outside the intended target region (see Fig. 2(f,l)).

### Quantification of targeting errors

The deviation of the actual electrode trajectory (defined by E_mri/rec-mri_, T_mri/rec-mri_; α_mri/rec-mri_, β_mri/rec-mri_; E_ct/rec-ct/obs-ct_, T_ct/rec-ct/obs-ct_; α_ct/rec-ct/obs-ct_, β_ct/rec-ct/obs-ct_), from the pre-operatively planned trajectory (E_plan_; T_plan_; α_plan_, β_plan_) was evaluated by the offsets T_mri/rec-mri/ct/rec-ct/obs-ct_ – T_plan_ and E_mri/rec-mri/ct/rec-ct/obs-ct_ – E_plan_ at target and entry point respectively along each of the three orthogonal directions (ML-AP-DV), by the Euclidean distances (ED) |T_mri/rec-mri/ct/rec-ct/obs-ct_ – T_plan_| and |E_mri/rec-mri/ct/ct-rec/obs-ct_ – E_plan_| and by the angular offsets α_mri/rec-mri/ct/rec-ct/obs-ct_ – α_plan_ (Δα_mri/rec-mri/ct/rec-ct/obs-ct_) and β_mri/rec-mri/ct/rec-ct/obs-ct_ – β_plan_ (Δβ_mri/rec-mri/ct/rec-ct/obs-ct_) in the ML and AP directions respectively. The offset at the target, as determined from histology, T_hist_ – T_plan_, was also computed and its correlation with MRI observations was tested using Pearson’s correlation coefficient (R, significance level = 0.05). All measures are expected to be near zero in case of no or minimal deviation between planned and actual trajectories. For instance, a negative offset would indicate that the deviation is more posterior (along AP), to the left (along ML) or to the ventral region (along DV) from the intended target location. The error measures were tested for statistical significance by a one-sided paired t-test against zero mean (significance level = 0.05). The group wise offsets at entry and target as measured from the segmentations directly in MRI/ CT, from the reconstructed trajectories in MRI/ CT and from the annotations by the human observer on CT were also compared against each other using a paired t-test (significance level = 0.05). All error measures are reported as mean ± s.d. For computation of error measures and statistics, we used Matlab (The Mathworks, Natick, MA, USA).

## Results

### Image fusion and alignment to stereotaxic atlas

**Figure 3:**
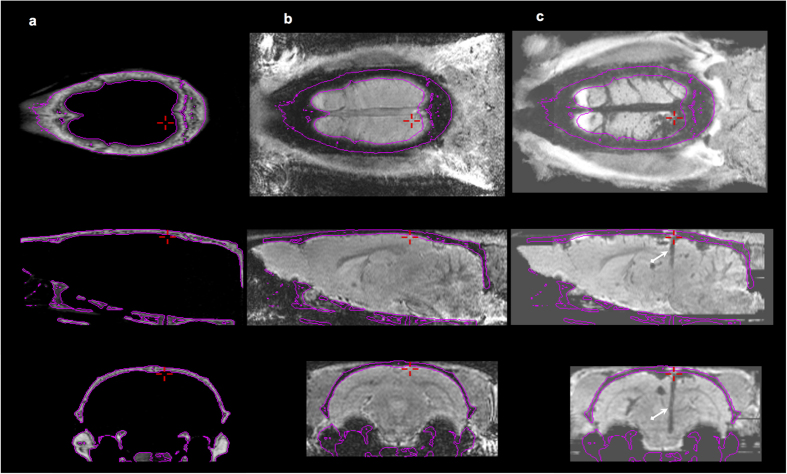
Multi-modal image fusion of pre- and post-operative images. Three orthogonal cross-sections (row 1: horizontal, row 2: sagittal, row 3: coronal) of fused pre-operative CT (**a**), pre-operative MR (**b**) and post-operative MR (**c**) images of the same animal. Registration quality can be visually appreciated from the iso-contours derived from CT corresponding to the skull and overlaid on the MR images (shown in pink). The cross-hair (+in red) indicates the entry point of the electrode trajectory. The trace of the trajectory is visible in the post-operative MRI as a trail of hypo-intense contrast (white arrows).

**Figure 4:**
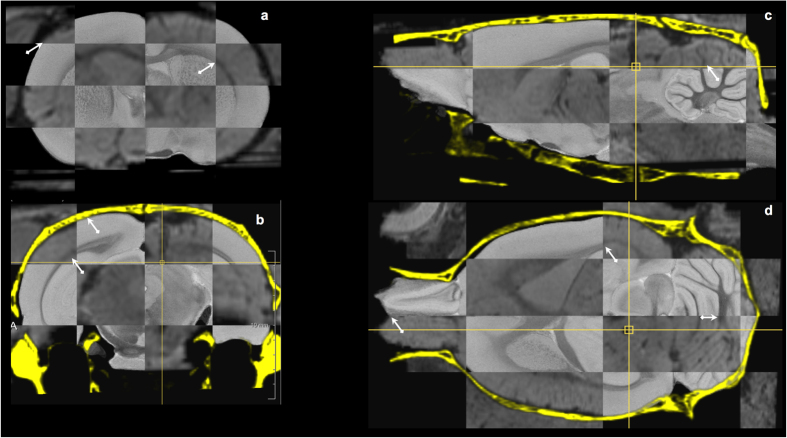
Image-to-template registration. Checkerboard visualization of corresponding coronal (**a**,**b**), sagittal (**c**) and horizontal (**d**) cross-sections of the *ex vivo* MRH atlas template^32^ and the spatially aligned and resampled *in vivo* post-operative MR image of a representative animal in our study, with the skull derived from its pre-operative CT overlaid (in yellow, panels **b**–**d**). Registration quality can be visually appreciated from the smooth transition between both images at tissue boundaries in different anatomical regions as indicated by the arrows (brain contour, corpus callosum, anterior commissure, white matter fissures of cerebellum).

The multi-modal image fusion of MRIpre, CTpre and MRIpost images of an individual animal is illustrated in Fig. 3(a–c). Correct alignment was visually verified in 3D by propagating the skull contours extracted from CTpre onto the other images and was confirmed for each animal. Likewise, the quality and overall consistency of the MRIpre to MRIpax registration for each animal was globally verified by visual inspection as illustrated in Fig. 4(a–d). Similar registration quality was also noted when MRIpost images were directly co-registered to MRIpax.

The overlay of CT on MRIpost in template space allowed comparing the alignment of the bregma-lambda (B-L) plane of the individual animal with that of the template (see Supplementary Figure S1(a,b)). The B-L distance as measured from CTpre was 8.31 ± 0.46 mm (mean ± s.d) and showed no significant difference (p > 0.05) compared to that of MRIpax (8.33 mm). While the maximum B-L distance was 9.20 mm, the minimum was 7.57 mm in this study population. When compared to the template, the offsets in the AP, ML and DV direction of the bregma points were significantly different from zero, while for lambda a significant offset was found both in the AP and the DV direction (see Table 1). The angular offset between the B-L plane of the template and that of the normalized images (i.e. Δγ) was significantly different from zero: 1.13 ± 1.33° (p < 0.005). These offsets present the anatomical variability in the study population and could also indicate the presence of residual mis-registration between the study images and the MRIpax template (discussed further).

### Adverse effects: Intracerebral hemorrhage

**Figure 5:**
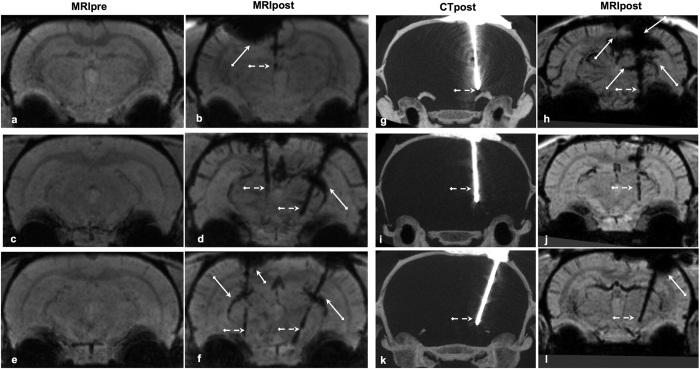
Surgery-related adverse effects visualized in post-operative MRI. Representative mIPs of pre-operative (column 1) MRI, mIPs of post-operative MRI (column 2, 4) and MIPs of post-operative CT (column 3 – maximum intensity projections) generated from coronal cross-sections covering the extent of the electrode trajectory. When compared to the pre-operative MR image (**a**,**c**,**e**), the post-operative images (**b**,**d**,**f**) show specific hypo-intense contrast along the trajectory (dotted white arrows), but often also a-specific hypo-intense clusters (indicated by continuous arrows) around the cortex (near electrode entry position) or superior colliculus vessel (radial to the trajectory), which appear to be likely indications of ICH or other deleterious effects of the surgery. As expected, post-operative CT in column 3 (**g**,**i**,**k**) does not show soft tissue contrast from the brain as well as any signs of deleterious effects as noted in the corresponding post-operative MRI (**h**,**j**,**l**).

Traces of ICH along the electrode trace were detected in 10 out of 23 cases (~40%, 7 out of 18 in Exp.1 and 3 out of 5 in Exp.2). Figure 5 compares the post-operative and pre-operative images of representative cases, in which an a-specific trail of hypo-intense contrast clusters can be explicitly observed. In particular, for trajectories targeting SNCD, hypo-intense signs of ICH on the T_2_-weighted post-operative MR images along the superior colliculus vessel network were noted (see Fig. 2(b,g)). Unlike in the clinic where the visualization of surgery related ICH is facilitated using CT, the pre-clinical CT imaging of the rodent brain not only lacks soft-tissue contrast, but also does not present any signs of hemorrhage. This is evident in Figs 2(g,h) and 5(g–l), where the MRIpost clearly presents signs of ICH, while the CTpost does not. Also, large deleterious effects in MRIpost could be unambiguously noted in post-operative images based on bilateral assessment as shown in Fig. 5. While in few cases comparing corresponding cross-sections in MRIpost with MRIpre helped to resolve ambiguous hypo-intense contrast variations along the electrode trajectory or elsewhere in the brain, based on our observations we believe that pre-operative MRI may not be always necessary (application dependent) to assess post-operative adverse effects.

### 3D trajectory reconstruction from MRI/CT

**Figure 6:**
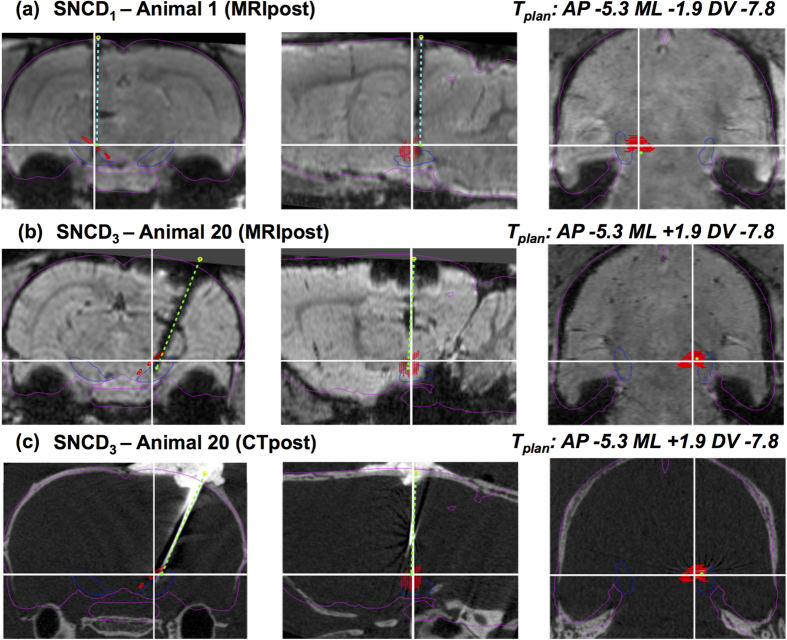
Reconstructed electrode trajectories from MRI and/or CT in individual animal. (**a**) Three orthogonal cross-sections of the post-operative MR image of animal A1 of group SNCD_1_ in MRIpax space (left: coronal, middle: sagittal, right: horizontal) at the planned target location (indicated by white cross-hairs) with the MRI-based reconstruction of electrode trajectory overlaid (dashed cyan line) and its entry and tip locations marked (E_rec-mri_: yellow o, T_rec-mri_: green •). The planned trajectory in this case coincides with the vertical cross-hair in the coronal and sagittal cross-sections. Contours of brain (magenta) and substantia nigra (blue) as derived from the MRH template^32^ are overlaid to illustrate the spatial normalization with this atlas. The contours of the target region (SNCD in the left hemisphere) as derived from the coronal slices of the atlas from Paxinos and Watson^8^ are depicted in red. The electrode tip location (T_rec-mri_) is more posterior than planned, but falls just inside the target region in this case; (**b**,**c**) Similar visualization as in (**a**), but for animal A20 of group SNCD_3_, in which SNCD in the right hemisphere was targeted with a planned ML inclination of the trajectory (~18°), followed by both post-operative MRI (**b**) and post-operative CT (**c**); The electrode trajectory reconstructed from its trace in post-operative MRI (**b**) visually matches well with the one reconstructed from post-operative CT with the electrode in place (**c**).

The hypo-intense contrast corresponding to the trace of the electrode trajectory could be manually delineated in each of the MRIpost images, except for one animal (Animal 8, Exp.1) for which the trace was insufficiently visible and which was therefore excluded from further analysis. Hence, in total 22 planned (E_plan_, T_plan_; α_plan_, β_plan_) and actual (E_mri/rec-mri_ E_mri/rec-mri_, T_mri/rec-mri_; α_mri/rec-mri_, β_mri/rec-mri_; E_ct/rec-ct/obs-ct_, T_ct/rec-ct/obs-ct_; α_ct/rec-ct/obs-ct_, β_ct/rec-ct/obs-ct_) trajectories in 19 animals were obtained as summarized in Supplementary Table S2. The reconstructed trajectories were visually verified on the MRIpost and/or CTpost images. Representative 3D electrode trajectories reconstructed from Animal 1 (Exp.1, left hemisphere, targeting SNCD_1_ at α_plan_ = 0°) and Animal 20 (Exp.2, right hemisphere, targeting SNCD_3_ at α_plan_ = −18°) are shown in Fig. 6(a–c). For Animal 1, although an offset at the entry point (E_plan_ vs E_mri_) and an inclination of the trajectory (Δα_mri/rec-mri_ = −0.69°/−0.73°; Δβ_mri/rec-mri_ = 0.51°/0.53°) can be perceived, the electrode tip landed within the target region. While targeting SNCD_3_ in Animal 20 (Exp.2), both translational (at target, ΔAP_mri/rec-mri/ct/rec-ct/obs-ct_ = 0.20/0.18/0.13/0.11/0.27 mm, ΔML_mri/rec-mri/ct/rec-ct/obs-ct_ = 0.19/0.49/0.17/0.5/0.13 mm, ΔDV_mri/rec-mri/ct/rec-ct/ct/obs-ct_ = −0.83/0.0/−0.83/0.0/−0.85 mm) and angular (Δα_mri/rec-mri/ct/rec-ct/obs-ct_ = 1.35°/1.35°/−0.45°/−0.44°/−0.87°; Δβ_mri/rec-mri/ct/rec-ct/obs-ct_ = 3.02°/2.84°/2.55°/2.37°/2.78°) offsets from the planned trajectory were noted in both modalities, but eventually the electrode tip was still within the target region (Fig. 6(b,c)).

#### *In vivo* assessment of targeting accuracy by MRI (Exp.1)

**Figure 7:**
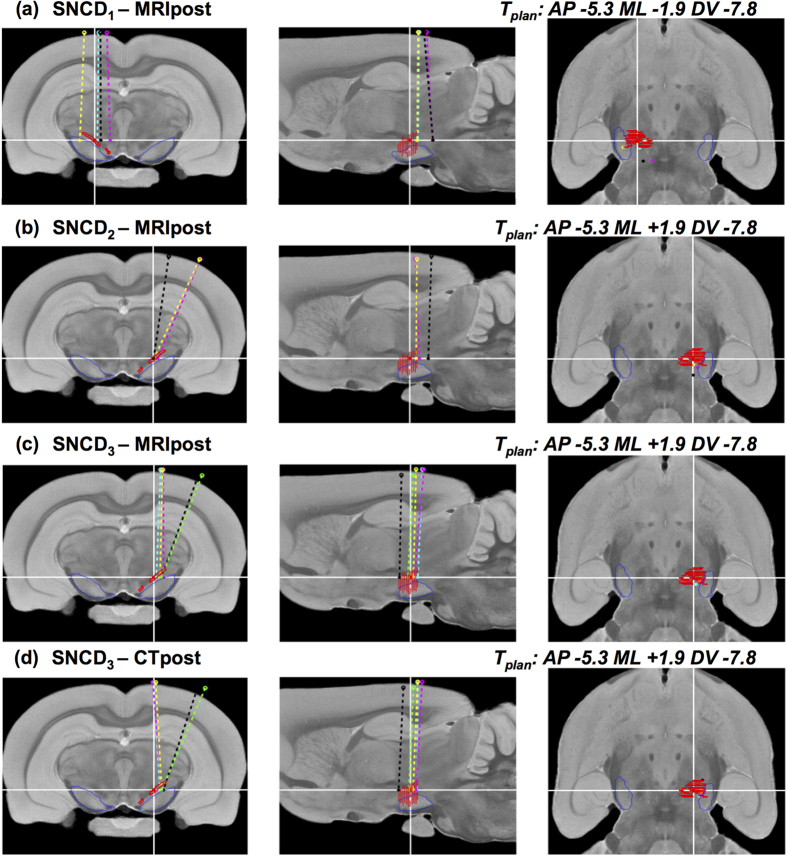
Reconstructed electrode trajectories for target SNCD. (**a**–**d**) Three orthogonal cross-sections of the MRH template at the planned surgical target SNCD with the reconstructed trajectories for all animals of group (**a**) SNCD_1_ (animals A1-4) – MRI only, (**b**) SNCD_2_ (animals A2-4) – MRI only, (**c**) SNCD_3_ (animals A16-20) - MRI and (**d**) SNCD_3_ (animals A16-20) - CT. In panel (a), the electrode tip locations for animals A2 (magenta), A3 (yellow) and A4 (black) all fall outside the target region, with two cases (animal A2 and A4) showing more than 2° anterior-posterior inclination. Panel (b) shows all animals of group SNCD_2_ (animal A2: magenta, A3: yellow, A4: black), where electrodes were planned at a medio-lateral inclination of the trajectory of 21°. Only the electrode tip location in animal A3 (yellow) falls within the target region, while the two others are more posterior. Panel (c) and (d) show the electrode trajectories for animals of Exp.2 (i.e. SNCD_3_ (A16-20)) as reconstructed from post-operative CT and MR images. All electrode tips in Exp.2 were within the intended target region of SNCD_3_. Both MRI and CT-based observations of electrode tip positions show mutual agreement, but a subtle ML difference could be noted at the entry point for some trajectories.

**Figure 8:**
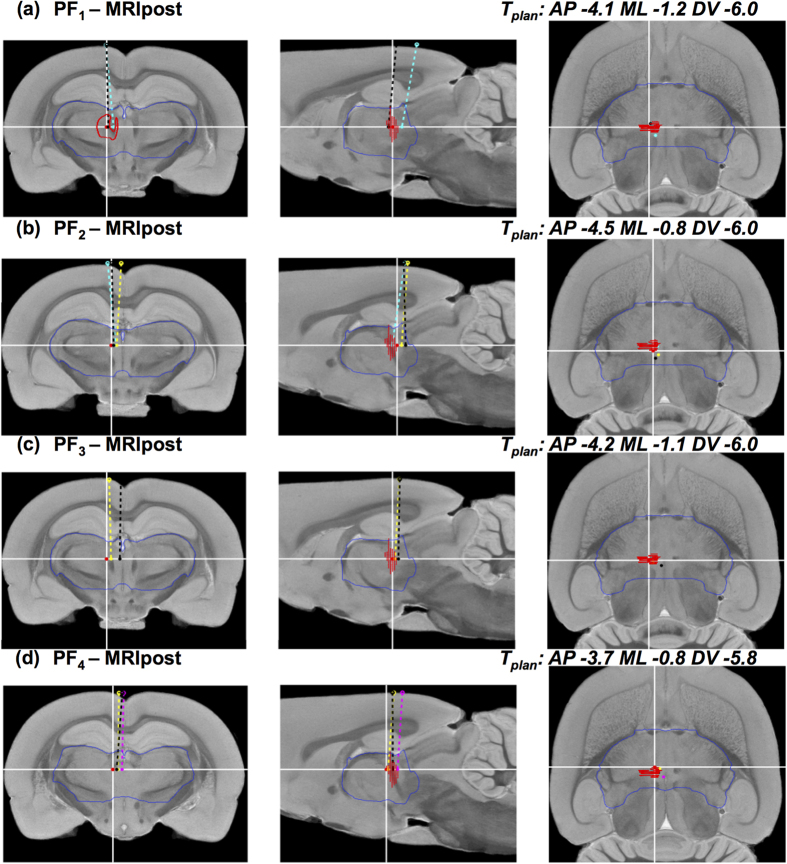
Reconstructed electrode trajectories for target PF. Similar visualizations as in Fig. 7, but for the trajectories targeting PF. Each row (**a**–**d**) shows coronal (left), sagittal (middle) and horizontal (left) cross-sections of the MRI template^32^ at the planned target location (indicated by white cross-hairs) with the reconstructed electrode trajectories overlaid for all animals for groups PF_1_ (**a**) PF_2_ (**b**) PF_3_ (**c**) and PF_4_ (**d**) respectively. The planned trajectory in each case coincides with the vertical cross-hair in the coronal and sagittal cross-sections. The contours of the target region (PF in the left hemisphere) as derived from the coronal slices of the Paxinos and Watson^8^ atlas are depicted in red and the contours of the diencephalon region (that encompasses PF) as derived from the MRH template^32^ are depicted in blue. In each group, the electrode tip location falls within the target region in one animal only, namely animal A6 in PF_1_ (black), A7 in PF_2_ (cyan), A11 in PF_3_ (yellow), and A15 in PF_4_ (black).

The variability in the trajectories in Exp.1 as determined from the post-operative MR images (MRIpost) for individual animals was examined in the common atlas space. Figure 7(a,b) summarizes the results for reconstructed trajectories (E_rec-mri_, T_rec-mri_) from groups SNCD_1_ and SNCD_2_, overlaid on the MRI template^32^. For SNCD_1_, 3 out of 4 electrode trajectories appeared to land outside the SNCD target region, with distances |T_rec-mri_ – T_plan_| from 0.6 mm up to 1.8 mm. This could be attributed to the inclination of these trajectories, laterally (Δα_mri/rec-mri_ = −0.29 ± 1.55°/−0.28 ± 1.64°, mean ± s.d.) and especially posteriorly (Δβ_mri/rec-mri_ = −1.43 ± 2.31°/−1.55 ± 2.47°). Similarly, for SNCD_2_, only 1 out of 3 trajectories appeared to land within the target region. In particular, the trajectory from Animal 4 was off-target by −1.2 mm in the AP direction and inclined by only −3.56° instead of the intended angle of α_plan_ = −18°, hence missing the target region completely. Figure 8(a–d) shows similar reconstructed trajectories for groups PF_1_ to PF_4_ overlaid on the MRI template of the atlas. In each of the sub-groups, only one trajectory was within the PF target and the ED offset was 0.56 ± 0.07 mm (mean ± s.d.). Although some electrode tips appear proximal to the target, the majority of electrodes were posterior and off in the ML direction.

**Figure 9:**
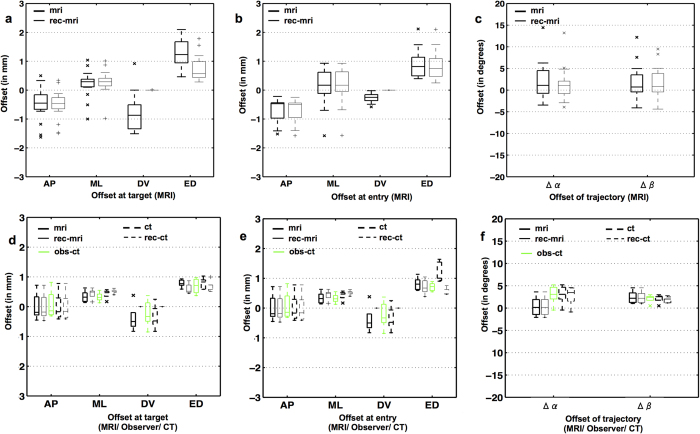
MRI/CT-based assessment of targeting accuracy. (**a**–**c**) Offsets in AP, ML and DV direction and Euclidean distance (ED) (in mm) between planned and actual target (**a**) and entry (**b**) locations as derived from post-operative MRI (mri) and its 3D trajectory reconstruction (rec-mri; n = 17 trajectories from Exp.1). (**c**) Offset in angular inclination (in degrees) between the planned and actual trajectory along ML (α) and AP (β) directions; (**d**–**e**) Translation and angular offsets at target and entry as computed from post-operative MR image (mri) and those that were reconstructed based on MRI (rec-mri) are illustrated as boxplots with continuous lines (n = 5 trajectories from Exp.2). Similar measures computed from post-operative CT image (ct) and the reconstruction from CT image (rec-ct) are shown as boxplots in dashed lines. The measurements from a manual observer in CTpost images (obs-ct) are illustrated in green colored boxplots.

Figure 9(a–c) and Table 2 summarize the observed deviations between the planned target and entry locations and the ones as determined from the post-operative MR images in Exp.1, either directly (T_mri_, E_mri_) or based on the reconstructed trajectory (T_rec-mri_, E_rec-mri_). For both observations, the deviations were significantly different from zero mean along the AP and ML directions for the target point and the entry point, but not along the DV direction. No significant difference in translational and angular offsets was noted between (T_mri_, E_mri_) and (T_rec-mri_, E_rec-mri_), except for the DV direction. The approximation of D_rec-mri_ = D_plan_ used for the reconstructed electrode tip localization (T_rec-mri_) resulted in near zero offsets (T_rec-mri_–T_plan_) in the DV direction, as expected. Angular offsets, Δα_mri/rec-mri_ and Δβ_mri/rec-mri_ along the ML and AP axes were 1.76 ± 4.35°/1.25 ± 3.95° (mean ± s.d.) and 1.80 ± 3.94°/1.77 ± 3.69° respectively. Both of these angular offsets were larger than 2° (in absolute value) in as much as 40% of the cases (~7 out of 17). The targeting accuracy computed from trajectories directly segmented from the post-operative MRI was comparable to that from the reconstructed trajectories, except that the latter ignored possible deviations in the DV direction (see p-values in Table 2). Overall, the extent of targeting variability in this study group can be summarized by an electrode positioning accuracy of about 1 mm (Euclidean distance offset), along with a trend of the electrode trajectory being inclined in the posterior-anterior direction (positive Δβ_mri/rec-mri_).

#### *In vivo* assessment of targeting accuracy by MRI and CT (Exp.2)

The electrode trajectories reconstructed from the segmentations of the electrode itself (from CTpost) or its trace (from MRIpost) in Exp.2 are qualitatively summarized in Fig. 7(c,d). In spite of minor angular offsets between MRIpost and CTpost observations (along ML direction in coronal view), all five trajectories (3 perpendicular, namely animal A16 cyan, A17 magenta, A18 yellow) and 2 inclined, namely animal A19 black, A20 green) resulted in the electrode tips landing within the intended SNCD_3_ target region. The corresponding quantitative measures of the targeting accuracy as illustrated in Fig. 8(d–f), confirmed this observation. At target, the offsets measured on the MRIpost (T_mri_–T_plan_ AP: 0.01 ± 0.46, ML: 0.34 ± 0.19; DV −0.39 ± 0.46 mm) showed good agreement with both CTpost (T_ct_–T_plan_ AP: 0.0 ± 0.48 mm, p = 0.97; ML: 0.42 ± 0.15 mm, p = 0.46; DV −0.35 ± 0.41 mm, p = 0.89) and localization by an observer in CTpost (T_obs_–T_plan_ AP: 0.08 ± 0.47 (p = 0.83), ML: 0.33 ± 0.16 (0.92); DV -0.23 ± 0.46 (p = 0.59) mm). While similar agreement between observations could be noted for the offset at the entry point along AP direction (MRI: −0.31 ± 0.56 mm vs CT/observer-CT: −0.25 ± 0.44 mm, p = 0.86/−0.16 ± 0.51 mm, p = 0.69), the offset at the entry point along ML direction as measured in MRIpost (0.35 ± 0.26 mm) deviates from both CTpost (−0.01 ± 0.14 mm, p = 0.03) and manual selection in CTpost (0 ± 0.19 mm, p = 0.04). Likewise, the deviation in the DV direction at the entry point in CTpost (CTpost: −1.09 ± 0.38 mm vs MRIpost: −0.37 ± 0.18 mm (p = 0.01) and observer: −0.5 ± 0.14 mm (p ≪ 0.001) could be attributed to the excessive exclusion (0.7 mm) of the dorsal regions of the segmentation during the electrode reconstruction step. Of these two, the deviation along ML direction in MRIpost directly impacts the angular offset along ML direction (Δα_mri_ = 0.36 ± 2.24° vs Δα_ct_ = 3.04 ± 2.18° (p = 0.09) and Δα_obs_ = 2.81 ± 2.24° (0.09)). Although this is clearly evident in the groupwise illustration of the electrode trajectories (angle of trajectories in the coronal cross-section of Fig. 7(c) and (d)), these differences between MRIpost, CTpost and observer were not statistically significant. The angular offset along AP direction (i.e. Δβ_mri_ = 2.43 ± 1.41° vs Δβ_ct_ = 2.16 ± 0.97° (p = 0.73) and Δβ_obs_ = 1.91 ± 0.73° (p = 1.0)) was also consistent across modalities.

As illustrated in Fig. 2(g–i), it should be noted that the segmentation of the electrode trace in MRIpost appears dilated compared to the physical electrode itself as perceived in CTpost. This may be caused by partial volume effects and/or by the enlargement of the trace during retraction of the electrode. Nevertheless, PCA based reconstruction of electrode trajectories from the trace showed excellent agreement with those of the CTpost and observer based recordings, especially at the tip, with a small discrepancy in ML direction at the entry point (<0.5 mm).

#### *Ex vivo* assessment of targeting accuracy by histology (Exp.1)

**Figure 10:**
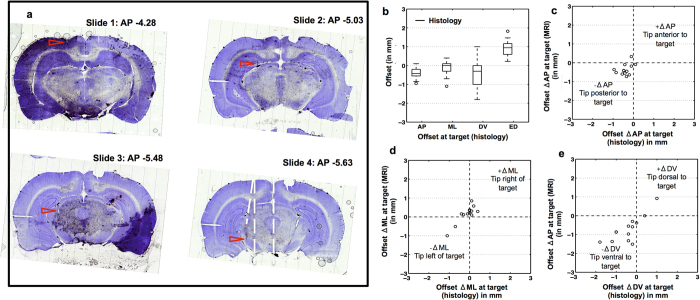
Histology-based assessment of targeting accuracy. (**a**) Sequence of 4 cresyl-violet stained coronal histological sections for animal A3, group SNCD_1_, at different AP distances from bregma (Paxinos −4.38 to −5.63 mm), showing the entry point (slide 1), the trajectory (slides 1–4) and the tip location (slide 4) of the electrode (red arrows). The actual tip location in this case was −0.33 mm off from the planned location and outside the targeted SNCD region. (**b**) Offsets in AP, ML and DV directions and Euclidean distance (ED) (in mm) between planned and actual target location as measured from post-operative histology for n = 14 electrode tip positions. (**c**–**e**) Correlation between offsets derived from histology and MRI show similar trends in AP (**c**), ML (d) and DV (**e**) directions. Positive correlation between MRI and histology observations was noted for offsets in ML (R = 0.87, p = 0.0001) and DV (R = 0.79, p = 0.001) directions.

The electrode tip coordinates T_hist_ as determined by histology are listed in Supplementary Table S2 for all animals of Exp.1. One animal (Animal 4) died at the end of the post-operative MRI session, hence histology could not be performed for this animal. The electrode tip could be located in 14 out of 16 cases for which histological images were available. A representative histology result for trajectory SNCD_1_ is illustrated in Fig. 10 for Animal 3 (Panel a). The trace of the electrode trajectory can be perceived in multiple cross-sections (from −4.28 to −5.63 mm AP), indicating that the trajectory was inclined in the AP direction or that sectioning was not parallel to mid-coronal plane. The electrode tip was determined to be outside the SNCD region. The tip location was off-target by a deviation (T_hist_ – T_plan_) in the AP direction of -0.33mm posterior. This is consistent with offsets (T_mri/rec-mri_ – T_plan_) in the AP direction of −0.45/−0.47 mm as observed with post-operative MRI. In general, MRI based (ED, 1.24 ± 0.50 mm (mean ± s.d.)) and histology based (0.92 ± 0.44 mm) quantification of targeting accuracy showed similar trends in targeting offsets (see Table 2, see Fig. 10(b)). When comparing the corresponding group-wise mean offsets along AP-ML-DV directions, only the offset along ML direction showed significant difference (pairwise two-tail t-test with significance = 0.05, yielded p < 0.05). Also, significant positive correlations between MRI and histology observations were noted along ML (R = 0.87, p = 0.0001) and DV (R = 0.79, p = 0.001) directions (see Fig. 10(c–e)).

### Variability in cranial landmarks and targeting accuracy

To determine whether the targeting inaccuracy that we quantified could be attributed to inter-animal variability in bregma/lambda, we assessed the correlation between the AP and ML offsets measured at the cranial landmarks in CTpre (Exp.1) and at the entry/tip locations in MRIpost (from Exp.1). Pearson’s correlation coefficient (R, significance level = 0.05) revealed a marginal positive linear dependence (R = 0.50, p = 0.03, see Supplementary Figure S1(B1)) between the AP offsets measured at bregma and entry point (E_mri/rec-mri_ – E_plan_), meaning AP differences between the bregma position of the individual animal and that of the template likely contributed to the observed deviations in the AP localization of the entry point. Such correlation was not found for the electrode tip, probably due to other confounding factors (e.g. skewed electrode) that may inconspicuously influence the trajectory and final tip position. Interestingly, we found a positive correlation (R = 0.55, p = 0.02, see Supplementary Figure S1(B2)) in the MRIpax space of the template between the angular offsets of the trajectories along the AP direction (β_mri/rec-mri_ – β_plan_) and those of the B-L plane as derived from pre-operative CT. This could indicate that the observed angular deviations in the AP direction may be in part attributed to deviations in the skull-flat position of the B-L plane in the MRIpax space after spatial normalization.

## Discussion

Previous studies on deep brain interventions in rodents already pointed out the issue of variability in stereotactic targeting accuracy^11^,^12^,^19^, resulting in a significant spread of the electrode tip within and outside the target region for different animals within the same study setup, despite identically defined entry and target points and using the same surgical procedure. These targeting inaccuracies may be caused by different factors, such as the stereotactic device, operator experience, inter-animal variability, the use of standard reference atlases, etc.^19^,^26^. If not properly assessed and accounted for, the variability in targeting accuracy could confound the results of the experiments relying on it. However, the current practice to assess targeting accuracy is based on histology, which has several limitations: it is performed *ex vivo* at end-point, it is destructive, and it is usually restricted to few 2D slices around the target region, thus failing to capture the complete 3D trajectory and possible signs of surgery-induced vascular damage along the trajectory.

We propose two workflows for non-invasive, *in vivo* imaging based assessment of targeting accuracy in the preclinical setting based on post-operative MR and/or CT imaging. The electrode trajectory is semi-automatically reconstructed in 3D from the images. By mapping the trajectories in different animals to the same atlas reference space after proper spatial normalization using image-to-atlas registration of the images, targeting accuracy can be objectively quantified for any chosen target. Our imaging-based evaluation of targeting accuracy for two common neuromodulation targets (SNCD, PF) was compared to conventional histology based assessment. Our study setup focused on the assessment of targeting accuracy, but did not involve actual electrode stimulations or recordings. Hence, retrospective investigation of the actual impact of targeting inaccuracies on neuromodulation experiments remained outside the scope of this work.

Our first experiment (Exp.1) mimics the typical setup of lesioning and needle insertion experiments in which the electrode is removed immediately after insertion. As the inserted electrode itself could not be imaged directly, we reconstructed its 3D trajectory from its hypo-intense trace in post-operative T_2_-weighted MRI (MRIpost). This trace was semi-automatically segmented and its main axis was determined by PCA and considered as the 3D orientation of the actual trajectory. Visual inspection showed that the hypo-intense contrast of the electrode trace might be ambiguous, especially around the entry and target points. Hence, their location along the reconstructed trajectory was not only derived from the images directly, but also inferred from the location of the dural surface in the atlas and the planned depth of the electrode.

In our second experiment (Exp.2), an alternative setup is considered in which the electrode is left in place, as is typically the case for actual neuromodulation studies like deep stimulation or recording. Hence, we investigated the feasibility of visualizing the implanted electrode directly using post-operative CT (CTpost). In addition, by subsequently removing the electrode and reconstructing its trajectory from post-operative MRI (MRIpost) as in Exp.1, we could compare both assessments (MRIpost vs CTpost) and provide validation for the MRIpost based assessment as performed in Exp.1. Our results demonstrate the feasibility of post-operative *in vivo* imaging with implanted electrode and show excellent agreement between the different assessments in this experiment with a relatively small sample size (Exp.2: n = 5).

While no significant difference between the bregma-lambda distance of our study population and that of the template was found, offsets along AP, ML and DV directions for each landmark individually were noted. Although this could indicate possible spatial normalization inaccuracies between the individual images and the atlas, proper global alignment between them was qualitatively verified for each animal individually based on visual assessment of corresponding anatomical features in the brain. Instead, the observed bregma/lambda offsets could also result from local inter-animal variability in the location of these cranial landmarks themselves^9^,^13^ and/or from ambiguity and uncertainty when visually locating bregma and lambda in the CT images. It should also be pointed out that, in our study setup, the location of bregma and lambda as derived from the CT images is independent from their location on the skull as determined during the surgery itself, as the CT was not used for surgery planning. Hence, observed offsets in the CT based bregma location as compared to its reference location in the atlas do not necessarily imply that a similar offset in the location of the actual versus the planned entry or target point is to be expected. Nevertheless, we found such correlation in our study for the entry point in AP direction.

Ideally, the B-L plane of each individual animal as derived from pre-operative CT should be perfectly horizontal after spatial normalization to the atlas in Paxinos space, which should be in skull-flat position. This was in general not the case in our study with a mean deviation of 1.13 ± 1.33° that was significantly different from 0 (p = 0.004). Such deviations could be caused by a gross global rotational error in the spatial normalization. However, as already mentioned above, this is unlikely as the registration of each individual image to the atlas was voxel based using information from the entire image (and not just few landmarks) and was carefully verified visually whereby no such apparent errors were noted. Another explanation may be that the template image itself that we used for spatial normalization to the Paxinos space, i.e. the MRH Wistar atlas^32^, is not perfectly skull-flat. This is not unlikely, as a similar deviation was observed for the MRH Sprague-Dawley rat atlas published by the same group^36^,^37^,^38^. Moreover, the 2D coronal contours of the target region that we obtained from Paxinos and Watson^8^ are not continuous in sagittal and horizontal planes. This complicates the correct 3D representation of these regions in the high-resolution isotropic MRH template, as evident from the missing contours of PF nucleus in the coronal cross-sections of Fig. 8(b–d). Resolving such discontinuities along the AP direction and establishing an accurate mapping of the subnucleus contours from Paxinos and Watson^8^ onto the 3D MRH template of Johnson *et al*.^32^ would be helpful.

The *ex vivo* assessment of targeting accuracy with histology-based measures relied on the ability to (manually and visually) overlay the histological slices of interest onto the corresponding cross-sections of the Paxinos atlas. However, the anatomical agreement between both may be compromised by tissue distortions occurring in the preparation of the slices and, more importantly, by possibly oblique sectioning of the specimen, i.e. along (near) coronal planes that are not guaranteed to be precisely parallel to the atlas planes. The trace of the electrode was often seen in multiple adjacent histological sections. Possible explanations could be that the animal was not exactly in skull flat position during surgery, that the electrode was inserted with incorrect orientation, that it got bended in AP direction during insertion, or that the specimen was sectioned obliquely at histology. This uncertainty further complicates the comparison of the 2D histology-based assessment of targeting accuracy with the 3D MRI-based assessment. Block face imaging that acquires a continuous series of adjacent sections with relatively minimal geometric distortion may be considered to alleviate this.

We report the targeting accuracy by the translational offsets of the actual electrode tip location with respect to the planned target location within the reference space of the atlas in which the target is originally defined, and by the angular offsets in the ML and AP inclination of the actual trajectory versus the planned trajectory. By co-localizing the actual electrode tip and the contours of the intended target region, we noted that only 30% of electrodes in Exp.1 landed within the target region (n = 17 trajectories). Although in Exp.2 all trajectories were on target (n = 5), translational and angular offsets from the pre-operative plan were observed in both experiments. These errors reflect the cumulative effect of various sources of inaccuracies, such as positioning inaccuracy while mounting the animal in the stereotactic device (e.g. incorrect skull flat positioning), uncertainty in localizing bregma (e.g. intra- and inter-observer variability), operator imprecision when positioning the electrode (e.g. limited accuracy of the device), accidental skewing of the electrode (e.g. when mounting the fragile electrode in the device or during insertion itself), spatial normalization inconsistencies (e.g. imperfect image-to-atlas registration), anatomical variability between the animals and the atlas used (e.g. inter-animal differences), etcetera. The proposed workflow allows to characterize the extent of targeting errors for a given target, trajectory, surgical procedure or operator. It could thus be used in future investigations to assess and minimize different sources of error, to compare and optimize different experimental setups, or to assess the benefits of animal-specific planning. With increased access to pre-clinical imaging facilities, it should become possible to exploit the full potential of *in vivo* imaging-based planning and assessment of stereotactic surgery in small animal models in larger scale studies.

In this study, both pre-operative and post-operative MR and CT imaging were used. However as demonstrated in Exp.2, post-operative imaging using either CT or MRI would be sufficient for establishing targeting accuracy in routine pre-clinical studies. We used CT to visualize the implanted electrode directly, but MRI could be used as well for this purpose provided that an MRI-compatible electrode and a sequence that minimizes susceptibility artifacts is used^39^,^40^,^41^,^42^. An advantage of post-operative MRI over CT is that MRI can provide additional information on possible deleterious effects of the surgery. By detecting the location of the electrode tip in the image and overlaying the contours of the target region after spatial normalization of the image to an appropriate atlas template, it becomes immediately evident whether the electrode tip is within or outside the target region. While Exp.2 demonstrated that post-operative MRI could be reliably co-registered to the MRH template directly (i.e. without making use of pre-operative MRI), the same is possible with post-operative CT, although a CT stereotaxic template could be beneficial for this purpose instead of the current skull-stripped MRI-based MRH template.

The image analysis workflow presented in this work focused on assessment of targeting accuracy, but could be readily extended towards animal-specific planning as well. For instance, additional pre-operative multi-modal and/or multi-parametric information, such as vasculature information derived from MR angiography images or, if such images are not available, from a vasculature atlas^43^ could be included. This information could be used prospectively, e.g. to determine the ‘optimal’ trajectory that avoids blood vessels and other critical structures and reduces the risk for deleterious effects resulting from the surgery, as well as retrospectively, e.g. to facilitate assessment of surgery induced vascular damage (e.g. ICH) from post-operative images. Likely evidence for ICH could be perceived in some animals in our study by the presence of traces of hypo-intense contrast in the T_2_-weighted post-operative MR images, which could be further investigated for optimal trajectory planning.

## Conclusion

We proposed a workflow for non-invasive *in vivo* imaging-based assessment of targeting accuracy in stereotactic neurosurgery in experimental rodent models, as an alternative for conventional *ex vivo* histology. The surgical trajectory and the actual target location are determined in 3D from post-operative images and their accuracy is quantitatively evaluated with respect to the planned target and trajectory as defined based on the standard Paxinos atlas. The targeting accuracy of stereotaxic brain surgery in this work was about 1 mm, with a trend of electrode tips being posterior to the target. The proposed workflow offers the benefit that deviations between the planned and actual trajectory can be detected and quantified non-invasively. Animals affected by possible confounding effects of the surgery, such as inaccurate targeting or vascular damage can be identified early on in the experiment. Pre-clinical studies relying on stereotactic neurosurgery can benefit by taking these confounds into account in the interpretation of the experiments.

## Additional Information

**How to cite this article**: Rangarajan, J. R. *et al*. Image-based *in vivo* assessment of targeting accuracy of stereotactic brain surgery in experimental rodent models. *Sci. Rep.* **6**, 38058; doi: 10.1038/srep38058 (2016).

**Publisher's note:** Springer Nature remains neutral with regard to jurisdictional claims in published maps and institutional affiliations.

## Supplementary Material

Supplementary Information

## Figures and Tables

**Figure 1:**
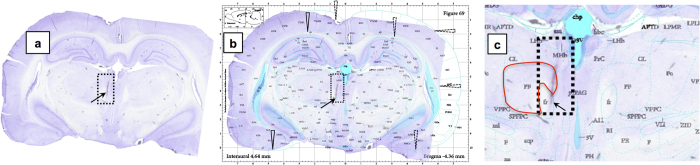
Electrode tip localization in a rat brain using histology. (**a**) Cresyl violet staining of a coronal histology section at the level of the target shows traces of the electrode trajectory (200 μm diameter) and its tip (dotted box, arrow); (**b**) Manual overlay of the histology section and the corresponding slice of the stereotaxic atlas^8^ with matching (continuous arrows) and non-matching (dotted arrows) regions indicated; (**c**) Zoomed-in version of (**b**), where the parafascicular nucleus (PF) that was targeted is highlighted (contour in red). The electrode tip landed outside the intended PF region.

**Table 1:** Offsets (mean ± s.d., in mm) between the coordinates of the cranial landmarks bregma and lambda as obtained from pre-operative CT and from the co-registered MRH template.

	AP	ML	DV	ED
Bregma (B)	−0.41 ± 0.42**	−0.12 ± 0.15*	−0.17 ± 0.14***	0.62 ± 0.18
Lambda (L)	−0.43 ± 0.33***	0.07 ± 0.13	−0.33 ± 0.19***	0.62 ± 0.25

AP anterior-posterior, ML medial-lateral, DV dorsal-ventral, ED Euclidean distance. ***p < 0.001; **p < 0.005; *p < 0.05.

**Table 2:** Offsets between the planned and actual target and entry points of the electrode trajectories determined from (a) segmentation of its trace in post-operative MRI, (b) a 3D reconstruction of the trajectory, (c) histology.

	T/E_mri_ – T/E_plan_ mean ± s.d. (in mm)			
	AP	ML	DV	ED
(a) MRI				
Target (T)	−0.50 ± 0.56**	0.21 ± 0.47*	−0.78 ± 0.61***	1.24 ± 0.50
Entry (E)	−0.67 ± 0.38***	0.13 ± 0.61*	−0.27 ± 0.16***	0.92 ± 0.47
	**β**_**mri**_ **– β**_**plan**_**°**	**α**_**mri**_ **– α**_**plan**_**°**	β_mri_ – β_plan_°*(max)*	α_mri_ – α_plan_°*(max)*
Angle	1.80 ± 3.94°*	1.76 ± 4.35°	12.20°	14.43°
**(b) Reconstructed**				
	**T/E**_**rec-mri**_ **– T/E**_**plan**_ **mean ± s.d. (in mm)**			
	**AP**	**ML**	**DV**	**ED**
Target (T)	−0.52 ± 0.51***	0.28 ± 0.42*	0.0 ± 0.0	0.76 ± 0.44
Entry (E)	−0.69 ± 0.38***	0.14 ± 0.61*	0.0 ± 0.0	0.87 ± 0.49
	**β**_**rec-mri**_ **– β**_**plan**_**°**	**α**_**rec-mri**_ **– α**_**plan**_**°**	β_rec-mri_–β_plan_°*(max)*	α_rec-mri_–α_plan_°*(max)*
Angle	1.77 ± 3.69°*	1.25 ± 3.95°	9.49°	13.21°
**(c) Histology**				
	**T**_**hist**_ **– T**_**plan**_ **mean ± s.d. (in mm)**			
	**AP**	**ML**	**DV**	**ED**
Target (T)	−0.42 ± 0.30***	-0.12 ± 0.39	−0.36 ± 0.72	0.92 ± 0.44
**Offset at target (T) – p value**				
	**ΔT**_**mri**_ **vs. ΔT**_**rec-mri**_	**ΔT**_**mri**_ **vs. ΔT**_**hist**_	**ΔT**_**rec**_ **vs. ΔT**_**hist**_	
AP	0.93	0.63	0.53	
ML	0.66	**0.04**	**0.01**	
DV	**0.00**	0.08	0.05	

See the text for methodological details. T target; E entry. AP anterior-posterior, ML medial-lateral, DV dorsal-ventral, ED Euclidean distance. ***p < 0.001; **p < 0.005; *p < 0.05.
