# Supplementary material for: Image-based in vivo assessment of targeting accuracy of stereotactic brain surgery in experimental rodent models
Source: Sci Rep. 2016 Nov 30;6:38058. doi: 10.1038/srep38058 (PMC5128925; doi:10.1038/srep38058)
Supplement: Supplementary Information [file srep38058-s1.pdf]

TITLE

Image-based *in vivo* assessment of targeting accuracy of stereotactic brain surgery in experimental rodent models

AUTHORS

Janaki Raman Rangarajan[1,2,*], Greetje Vande Velde[2,3], Friso van Gent[3,4], Philippe De Vloo[4], Tom Dresselaers[2,3], Maarten Depypere[1], Kris van Kuyck[4], Bart Nuttin[4], Uwe Himmelreich[2,3], Frederik Maes[1]

(* corresponding author janaki.rangarajan@kuleuven.be)

AFFILIATIONS

[1]Department of Electrical Engineering (ESAT/PSI), KU Leuven & Medical Imaging Research Center, University Hospital Leuven, Leuven, Flanders, Belgium

[2]Molecular Small Animal Imaging Center (MoSAIC), Faculty of Medicine, KU Leuven, Leuven, Flanders, Belgium

[3]Biomedical MRI unit, Department of Imaging and Pathology, Faculty of Medicine, KU Leuven, Leuven, Flanders, Belgium

[4]Laboratory for Experimental Functional Neurosurgery, Department of Neurosciences, Faculty of Medicine, KU Leuven, Leuven, Flanders, Belgium

SUPPLEMENTARY TABLES

Supplementary Table S1: Neuromodulation targets and planned electrode trajectories in each animal defined according to the stereotaxic rat brain atlas of Paxinos and Watson[8]. Coordinates in mm relative to bregma (0,0,0). The entry point is located on the dural surface. AP anterior-posterior, ML medial-lateral, DV dorsal-ventral.

**Animal**	**Target**	**Target point (T_{plan})**			**Probe angle**		**Entry point (E_{plan})**			**Depth (D_{plan})**
		AP	ML	DV	α_{plan}	β_{plan}	AP	ML	DV	
**Exp. 1**										
A1	$SNCD_1$	-5.3	-1.9	-7.8	0º	0º	-5.3	-1.9	-0.2	7.6
A2	$SNCD_1$	-5.3	-1.9	-7.8	0º	0º	-5.3	-1.9	-0.2	7.6
A3	$SNCD_1$	-5.3	-1.9	-7.8	0º	0º	-5.3	-1.9	-0.2	7.6
A4	$SNCD_1$	-5.3	-1.9	-7.8	0º	0º	-5.3	-1.9	-0.2	7.6
A2	$SNCD_2$	-5.3	+1.9	-7.8	-21º	0º	-5.3	-5.0	-0.8	7.6
A3	$SNCD_2$	-5.3	+1.9	-7.8	-21º	0º	-5.3	-5.0	-0.8	7.6
A4	$SNCD_2$	-5.3	+1.9	-7.8	-18º	0º	-5.3	-4.5	-0.6	7.6
A5	PF_1	-4.1	-1.2	-6.0	0º	0º	-4.1	-1.2	-0.2	5.8
A6	PF_1	-4.1	-1.2	-6.0	0º	0º	-4.1	-1.2	-0.2	5.8
A7	PF_2	-4.5	-0.8	-6.0	0º	0º	-4.5	-0.8	-0.2	5.8
A8	PF_2	-4.5	-0.8	-6.0	0º	0º	-4.5	-0.8	-0.2	5.8
A9	PF_2	-4.5	-0.8	-6.0	0º	0º	-4.5	-0.8	-0.2	5.8
A10	PF_2	-4.5	-0.8	-6.0	0º	0º	-4.5	-0.8	-0.2	5.8
A11	PF_3	-4.2	-1.1	-6.0	0º	0º	-4.2	-1.1	-0.4	5.6
A12	PF_3	-4.2	-1.1	-6.0	0º	0º	-4.2	-1.1	-0.4	5.6
A13	PF_4	-3.7	-0.8	-5.8	0º	0º	-3.7	-0.8	-0.4	5.4
A14	PF_4	-3.7	-0.8	-5.8	0º	0º	-3.7	-0.8	-0.4	5.4
A15	PF_4	-3.7	-0.8	-5.8	0º	0º	-3.7	-0.8	-0.4	5.4
**Exp. 2**										
A16	$SNCD_1$	-5.3	+1.9	-7.8	0º	0º	-5.3	+1.9	-0.2	7.6
A17	$SNCD_1$	-5.3	+1.9	-7.8	0º	0º	-5.3	+1.9	-0.2	7.6
A18	$SNCD_1$	-5.3	+1.9	-7.8	0º	0º	-5.3	+1.9	-0.2	7.6
A19	$SNCD_2$	-5.3	+1.9	-7.8	-18º	0º	-5.3	+4.7	-0.8	7.6
A20	$SNCD_2$	-5.3	+1.9	-7.8	-18º	0º	-5.3	+4.7	-0.8	7.6

Supplementary Table S2: Actual electrode trajectories determined from (a, d) direct segmentation in post-operative *in vivo* MRI, (b) 3D reconstruction of the trajectory, (c) *ex vivo* histology, (e) direct segmentation in CTpost and (f) observer in CTpost. Coordinates are defined in millimeters (mm) relative to bregma (0,0,0) and angles in degrees (º). --: electrode trace not discernable; n.a.: data not available. AP anterior-posterior, ML medial-lateral, DV dorsal-ventral.

**Animal**	**Target**	**(a) MRI**								**(b) Reconstructed**				**(c) Histology**			
		**Tip (T_{mri})**			**Probe angle**		**Entry (E_{mri})**			**Tip ($T_{rec-mri}$)**			**On target?**	**Tip (T_{hist})**			**On target?**
		AP	ML	DV	α_{mri}	β_{mri}	AP	ML	DV	AP	ML	DV		AP	ML	DV	
A1	SNCD$_1$	-5.82	-1.74	-8.66	-0.70º	0.51º	-5.90	-1.64	-0.69	-5.84	-1.73	-7.80	Yes	-6.15	-2.0	-8.80	No
A2	SNCD$_1$	-6.85	-0.86	-8.76	1.51º	-2.62º	-6.13	-1.10	-0.43	-6.77	-0.89	-7.80	No	--	--	--	n.a.
A3	SNCD$_1$	-5.75	-2.90	-8.39	-2.19º	0.50º	-5.56	-2.60	-0.62	-5.77	-2.88	-7.80	No	-5.63	-3.0	-8.00	No
A4	SNCD$_1$	-6.93	-1.50	-8.68	0.20º	-4.09º	-5.98	-1.53	-0.55	-6.80	-1.50	-7.80	No	n.a.	n.a.	n.a.	n.a.
A2	SNCD$_2$	-5.90	+2.00	-8.35	-16.5º	-1.99º	-5.65	+4.88	-1.05	-5.92	+2.21	-7.80	No	-5.85	+2.00	-8.20	Yes
A3	SNCD$_2$	-5.71	+1.39	-9.17	-15.1º	0.31º	-5.76	+4.89	-0.90	-5.71	+1.97	-7.80	Yes	-5.78	+1.20	-9.00	No
A4	SNCD$_2$	-6.48	+1.75	-9.17	-3.56º	1.42º	-6.71	+2.92	-0.63	-6.49	+1.93	-7.80	No	n.a.	n.a.	n.a.	n.a.
A5	PF$_1$	-4.83	-0.83	-5.08	6.27º	12.2º	-5.62	-1.21	-0.60	-4.71	-0.75	-5.99	No	-4.44	-1.20	-5.00	No
A6	PF$_1$	-3.77	-1.08	-6.38	1.10º	4.99º	-4.32	-1.20	-0.53	-3.84	-1.09	-6.00	Yes	-4.20	-1.50	-6.00	Yes
A7	PF$_2$	-4.00	-0.44	-6.86	4.66º	7.58º	-4.96	-1.00	-0.50	-4.16	-0.51	-6.00	Yes	--	--	--	n.a.
A8	PF$_2$	--	--	--	--	--	--	--	--	--	--	--	n.a.	-4.92	-1.20	-5.60	No
A9	PF$_2$	-4.68	-0.51	-7.33	-2.58º	3.46º	-5.14	-0.18	-0.78	-4.79	-0.44	-6.00	No	-4.60	-0.40	-6.40	No
A10	PF$_2$	-5.05	-0.64	-6.00	1.24º	-1.19º	-4.93	-0.77	-0.22	-5.05	-0.64	-6.00	No	-4.92	-1.20	-5.60	No
A11	PF$_3$	-4.30	-0.79	-6.97	1.11º	2.53º	-4.64	-0.93	-0.56	-4.36	-0.81	-6.00	Yes	-4.63	-1.0	-6.40	No
A12	PF$_3$	-4.56	-0.25	-7.51	-0.57º	0.71º	-4.66	-0.17	-0.75	-4.58	-0.23	-6.00	No	-5.15	-1.0	-6.20	No
A13	PF$_4$	-4.34	-0.22	-7.20	-0.85º	2.75º	-4.73	-0.10	-0.64	-4.43	-0.19	-5.80	No	-3.90	-0.60	-7.60	Yes
A14	PF$_4$	-3.79	-0.46	-6.10	-0.65º	3.74º	-4.17	-0.40	-0.58	-3.82	-0.46	-5.80	No	-3.60	-0.80	-6.00	No
A15	PF$_4$	-4.12	-0.60	-6.67	-3.46º	-0.15º	-4.10	-0.18	-0.54	-4.11	-0.54	-5.80	Yes	-4.18	-0.80	-6.80	Yes
		**(d) MRI**								**(e) CT (Tip T_{ct})**				**(f) CT observer (Tip T_{obs-ct})**			
A16	SNCD$_2$	-5.75	+2.04	-8.40	-2.08º	2.21º	-6.06	+2.34	-0.32	-5.71	+2.31	-7.97	Yes	-5.56	+2.16	-7.75	Yes
A17	SNCD$_2$	-5.52	+2.53	-8.19	0.14º	4.53º	-6.11	+2.51	-0.71	-5.62	+2.47	-8.31	Yes	-5.61	+2.45	-8.13	Yes
A18	SNCD$_2$	-5.49	+2.31	-7.42	-1.21º	1.34º	-5.65	+2.46	-0.66	-5.47	+2.40	-7.55	Yes	-5.44	+2.21	-7.43	Yes
A19	SNCD$_2$	-4.58	+2.22	-8.30	-14.4º	1.03º	-4.71	+4.71	-0.84	-4.52	+2.37	-8.28	Yes	-4.48	+2.28	-8.17	Yes
A20	SNCD$_2$	-5.10	+2.09	-8.63	-16.6º	3.02º	-5.50	+4.85	-1.14	-5.17	+2.07	-8.63	Yes	-5.03	+2.03	-8.65	Yes

SUPPLEMENTARY FIGURES

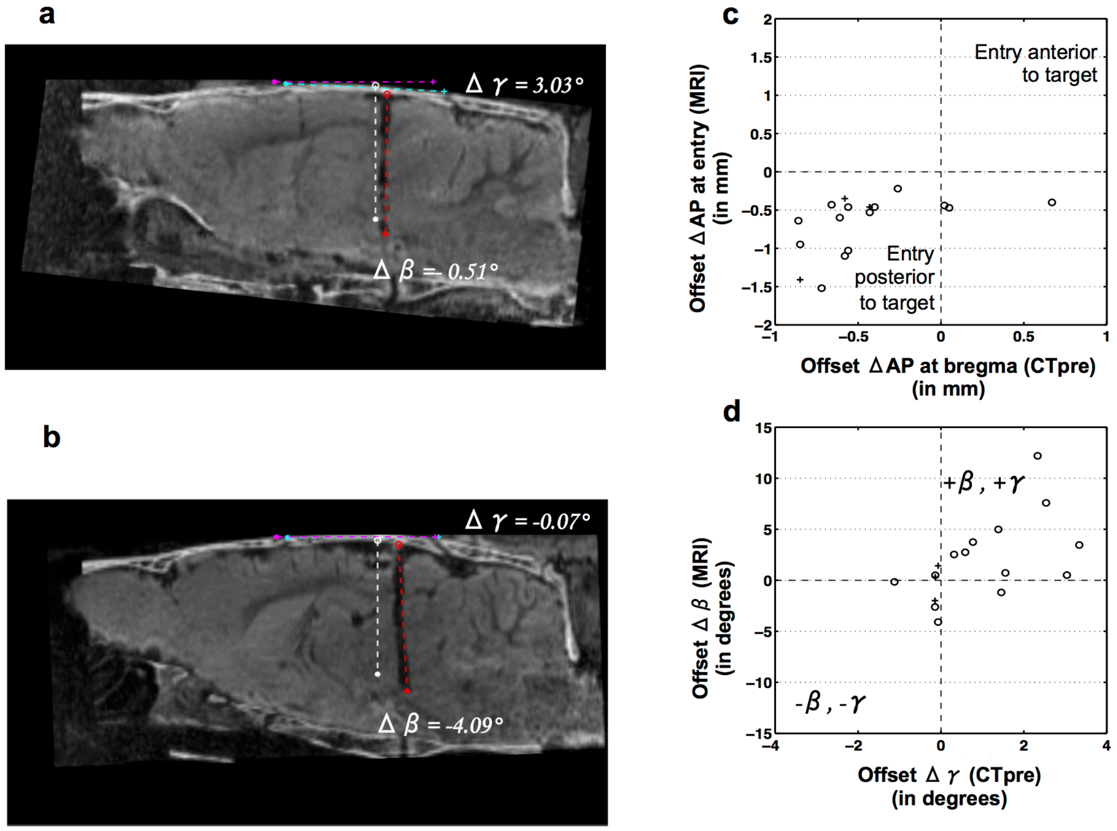

Supplementary Figure S1: Skull flat orientation and cranial landmarks of template versus study image. (a, b): Sagittal cross-section (mIP) of fused pre-operative CT and post-operative MR images at the actual electrode tip location for animal A1 (panel a, ML = -1.74) and for animal A4 (panel b, ML = -1.50), after spatial normalization to the MRH template of Johnson *et al.*[32]. Planned (dashed line in white) and actual (dashed line in red) electrode trajectories as determined from MRI are shown, as well as the projected locations of cranial landmarks bregma (marked with ◦) and lambda (+) as derived from the template (in magenta) and from the CT image of the animal itself (in cyan). In both cases, the bregma landmark derived from CT is located posterior to that of the template. In (a), the bregma-lambda (B-L) plane of the animal (dashed line in cyan) shows angular deviation of about 3° from the (horizontal) B-L plane of the template (dashed line in magenta), while in (b), nearly no angular offset between both planes can be noted; (c-d)**:** (c) Correlation analysis of experiment 1, revealed positive correspondence between the offsets at the entry location measured from MRI and the bregma position measured from pre-operative CT, but only along AP direction (R = 0.50, p-value = 0.03). A negative ΔAP means that the electrode entry location is posterior to the intended planned entry position. (d) Angular deviation of the electrode trajectory along AP direction measured from MRI correlates with the deviation from skull flat position measured from pre-operative CT (R = 0.55, p-value = 0.02). Trajectories in right hemisphere are marked with + in (c, d).
